# Prevalence of Unmet Service Needs and Associated Person‐Reported Outcomes in Long‐Term Services and Supports in the United States

**DOI:** 10.1111/jgs.70551

**Published:** 2026-06-19

**Authors:** Romil R. Parikh, Jack M. Wolf, Nishka U. Shetty, Ashwin A. Pillai, Benjamin W. Langworthy, Chanee D. Fabius, Stephanie Giordano, Eric Jutkowitz, Tetyana Shippee

**Affiliations:** ^1^ Division of Health Policy & Management University of Minnesota School of Public Health Minneapolis Minnesota USA; ^2^ Department of Biostatistics, Epidemiology, and Informatics University of Pennsylvania Perelman School of Medicine Philadelphia Pennsylvania USA; ^3^ Kymera Medical Group Eastern New Mexico Medical Center Roswell New Mexico USA; ^4^ Division of Cardiology Virginia Commonwealth University Richmond Virginia USA; ^5^ Division of Biostatistics and Health Data Science University of Minnesota School of Public Health Minneapolis Minnesota USA; ^6^ Division of Health Policy & Management Johns Hopkins Bloomberg School of Public Health Baltimore Maryland USA; ^7^ Human Services Research Institute Cambridge Massachusetts USA

**Keywords:** health‐related social needs, long‐term care, LTSS quality, Medicaid, patient‐reported outcomes

## Abstract

**Background:**

Long‐term services and supports (LTSS) serve > 9 million adults in the United States. We examined prevalence and quality of life (QoL)‐related outcomes of consumer‐reported unmet LTSS needs in publicly‐funded LTSS.

**Methods:**

We pooled cross‐sectional data from the 2016–2017, 2017–2018, 2018–2019, 2021–2022, and 2022–2023 National Core Indicators–Aging and Disability Adult Consumer Surveys. We included 61,829 adults (34% with age < 65 years; 66% female; 27 states). Survey weights generated population‐representative estimates. We ascertained unmet LTSS needs by response to: “Do the long‐term care services you receive meet your current needs and goals?” (dichotomized‐ yes vs. no). We estimated weighted prevalence of unmet LTSS needs overall and across subpopulations, and examined associations with two self‐reported QoL‐related outcomes (dichotomized‐ yes/no): (1) being active in community; (2) satisfied with how one spends one's day. Prevalence ratios (PRs) were estimated using weighted Poisson regression with robust variance estimators, adjusting for demographics, health and functional status, multimorbidity, funding program, residence setting, state, and survey year.

**Results:**

Among respondents, 18,004 (weighted‐estimate, 29%) reported unmet LTSS needs. Prevalence of unmet needs varied across sociodemographic strata, care settings (higher in community‐based than in residential‐care settings), and funding programs (lower in PACE than in Medicaid programs). Individuals with unmet needs were significantly less likely to report being active in the community (PR, 0.65; 95% CI, 0.62–0.68) and satisfied with how they spend their day (PR, 0.62; 95% CI, 0.60–0.64). Post hoc analyses revealed potential dose–response associations between increasing degree of unmet LTSS needs and outcomes.

**Conclusions:**

Consumer‐reported unmet LTSS needs are frequent, vary greatly across care settings and funding programs, and are associated with poorer QoL‐related outcomes; highlighting substantial system‐level gaps in the fragmented LTSS landscape and the need for coordinated investments and structural reforms to better meet the needs of individuals relying on these services.

## Summary

- Key Points
  ○ Nearly one‐third of adults receiving long‐term services and supports (LTSS) reported unmet needs, with substantial variation across care settings, funding programs, and individual characteristics.
  ○ Unmet LTSS needs were consistently associated with poorer person‐reported outcomes, including lower community participation and reduced satisfaction with daily activities.
  ○ These patterns suggest that unmet needs reflect systemic gaps in a fragmented LTSS ecosystem rather than isolated deficiencies within specific programs.
- Why Does This Paper Matter?
  ○ This study provides population‐level evidence that unmet LTSS needs are both common and consequential for individuals' daily lives, and vary greatly across care settings and funding programs, underscoring the limitations of the current patchwork system. The findings highlight the need for greater investment and coordinated structural reforms to improve LTSS delivery and outcomes for aging and disabled populations.

## 1 Introduction

In 2022, Medicaid long‐term services and supports (LTSS) served 9.1 million adults, with total expenditure exceeding $200 billion [1]. LTSS encompass a broad range of health, personal care, and support services to older adults and people with disabilities; for example, skilled nursing facility‐based care, nursing home care, home healthcare, personal care, transportation, meal delivery, adult day care, home modifications, respite care, or assistance with daily activities [2, 3]. LTSS are provided across home‐ and community‐based, congregate, and residential care settings such as nursing facilities [1]. Despite the central role of LTSS in maintaining safety, function, and daily life, many users report unmet needs for services [3]. However, there is sparse population‐level evidence characterizing how unmet service needs vary across different care settings and funding mechanisms.

Furthermore, unmet needs in different types of LTSS have been linked to potentially preventable health care utilization and diminished well‐being [4, 5, 6, 7, 8]. However, evidence linking unmet LTSS needs with poor outcomes remains limited by lack of generalizable population‐representative estimates, reliance on small or single‐state samples, heterogeneous definitions of unmet need, and a lack of standardized, person‐centered quality of life (QoL) measures [4, 5, 6, 7, 8]. As a result, it remains unclear whether the prevalence and consequences of unmet LTSS needs differ meaningfully across diverse service delivery and financing contexts. Knowing whether unmet LTSS needs are associated with poorer outcomes is also relevant for informing resource allocation and policy‐making for LTSS in the United States, for which the consequences of unmet LTSS needs have not yet been quantified on a national scale [1, 2, 3, 4, 5, 6, 7, 8].

Using nationally pooled data from the National Core Indicators–Aging and Disability (NCI‐AD) adult consumer surveys (ACS) spanning 27 states, we examined the associations of overall unmet LTSS needs with person‐reported QoL‐related outcomes and healthcare utilization among adults receiving LTSS. The NCI‐AD ACS is a unique survey that measures person‐reported quality in publicly funded LTSS (through Medicaid and other waivers) and includes related administrative as well as consumer‐reported data [3, 4]. We leveraged serial cross‐sectional survey data from the NCI‐AD ACS because, to our knowledge, this is the largest and the only data source for measuring consumer‐reported unmet LTSS needs with a single, direct question to LTSS users in the United States [3, 4]. We hypothesized that unmet LTSS needs would have a high prevalence, its prevalence would vary across different subpopulations, and it would be associated with poorer person‐centered outcomes.

## 2 Methods

The reporting of this cross‐sectional study followed the strengthening the reporting of observational studies in epidemiology (STROBE) reporting guideline. This study involves secondary data analyses of de‐identified survey data and was deemed exempt by the Institutional Review Board at the University of Minnesota.

### 2.1 Study Design and Data Source

We conducted a cross‐sectional study using pooled data from the 2016–2017, 2017–2018, 2018–2019, 2021–2022, and 2022–2023 survey waves of the NCI‐AD ACS. The NCI‐AD ACS is an annual survey administered jointly by the Human Services Research Institute, ADvancing States, and participating state governments, assessing the experiences, service needs, health, and well‐being of adults receiving publicly funded LTSS [3, 4]. Surveys are completed in person, over the phone, or online, and administered by trained interviewers to consumers or their proxies, and include standardized items on functioning, service access, and QoL. All older adults receiving at least one active service (not including case management) through publicly funded LTSS are eligible for inclusion in the NCI‐AD ACS. State participation is voluntary; participating states set their sampling frame from different programs such as Medicaid aging & disability waiver or managed LTSS, with the requirement for maintaining < 5% margin of error and a minimum of 400 surveys in each wave. The NCI‐AD generates sampling weights which account for state‐specific sampling frames to arrive at aggregated, national estimates. A total of 27 states contributed at least one survey wave in our sample between 2016 and 2023. More details about the conduct of NCI‐AD ACS are provided in Supporting Information.

### 2.2 Study Population

The pooled survey data from 2016 to 2023 included 69,444 respondents (Figure S1). We excluded 7533 respondents who were missing response to the question for unmet LTSS needs, 73 respondents living in homeless or temporary shelter settings, and 9 respondents who reported a gender category other than male or female (due to very small numbers and resultant lack of generalizability), resulting in a final analytic sample of 61,829 adult LTSS users.

### 2.3 Exposure

The primary exposure was overall unmet LTSS needs, measured using a single self‐reported survey item. We identified respondents with self‐reported unmet LTSS needs by examining responses to a single self‐reported survey item, “Do the long‐term care services you receive meet your current needs and goals?” Responses to the question included “yes”, “some”, and “no”. In primary analyses, participants who responded “no” or “some” were categorized as having overall unmet LTSS needs. Responses were coded dichotomously as any unmet need versus no unmet need.

### 2.4 Outcomes

We examined two QoL‐related community living outcomes, each coded yes/no: (1) Being active in the community from the question “Are you as active in your community as you'd like to be?” (e.g., church groups and book club); (2) Satisfaction with how one spends one's day ascertained by the question: “Do you like how you usually spend your time during the day?” These measures were selected due to their relevance for person‐centered care, quality monitoring, and LTSS program evaluation, and because they are consistently collected across all survey waves. The person‐reported outcomes were asked only in the consumer section of the survey, not allowing for a proxy response.

### 2.5 Covariates

Covariates were selected a priori based on known associations with LTSS needs, service utilization, or functional outcomes [3, 4, 5, 6, 7, 8]. These included age, sex, race and ethnicity, rurality (following rural–urban continuum area classification codes based on ZIP codes; includes metropolitan, micropolitan, small towns, and rural areas); physical disability (yes/no), intellectual or developmental disability (yes/no), history of brain injury (yes/no), mental health diagnosis (including schizophrenia, bipolar disorder, or major depressive episode; yes/no), known diagnosis of Alzheimer's disease or related dementias (ADRD), self‐rated health (measured on a 5‐level Likert scale ranging from poor to excellent), multiple chronic diagnoses (> 1 chronic disease), assistance needs for self‐care (response to question: “How much assistance with self‐care do you generally need? Things like bathing, dressing, going to the bathroom, eating or moving around your home/where you live.”; categorized as no, some, or a lot), living arrangement, residence setting (community, assisted living, nursing facility, or other), LTSS funding program (Medicaid HCBS waiver for aging & disability in a fee‐for‐service system, managed LTSS, nursing facility, Older Americans Act, Program of All‐Inclusive Care for the Elderly, or other), Medicare status (yes/no), proxy response indicator, survey wave, and state identity indicators.

### 2.6 Statistical Analyses

We first summarized participant characteristics overall and stratified by self‐reported unmet LTSS needs. Weighted means and proportions were estimated using NCI‐AD survey weights to reflect state‐level LTSS populations. We summarized weighted prevalence of unmet LTSS needs (applying NCI‐AD survey weights), stratified by participants' demographic, social, health, and functional status. Next, we estimated the prevalence of each outcome by estimating the weighted proportion of LTSS users reporting an outcome event separately for individuals with and without self‐reported unmet LTSS needs. We then estimated adjusted prevalence ratios (PRs) for each of two outcomes using survey‐weighted Poisson regression models with robust “sandwich” variance estimators. Models adjusted for age, sex, race and ethnicity, disability type, mental health and physical health diagnoses, self‐rated health, level of assistance needed for self‐care, marital status, multiple chronic diagnoses, living arrangement, residence setting, LTSS funding program, Medicare enrollment, proxy response, and survey year, with fixed effects for state to account for between‐state programmatic variation. Adjusted associations were also estimated within the following pre‐specified subgroups via stratification: no known dementia diagnosis, dementia diagnosis, age < 65 years, age ≥ 65 years, and Medicaid recipient. When modeling, we included a “missing” level for all covariates with missing data. Description of the NCI‐AD survey weights are in Supplemental Methods in Supporting Information. Missing data ranged from 0% to 9.2% per covariate (mean, 1.99% per covariate).

We conducted two sensitivity analyses; first, in which we used augmented inverse probability weighting to compute doubly robust estimators (see Supplemental Methods in Supporting Information), and the second in which we repeated primary analyses after excluding respondents from nursing facilities. We also estimated association in the subgroup of nursing home residents. We conducted post hoc secondary analyses in which we evaluated the potential for a dose–response association by analyzing the exposure variable of consumer‐reported unmet LTSS needs as a three‐level variable with levels “yes”, “some”, or “none”. We refit the primary regression models using the three‐level exposure for unmet LTSS needs. From these models, we estimated covariate‐adjusted PRs as well as marginal prevalences for each outcome by level of unmet need, using survey weights to generate population‐representative estimates. Additionally, we reported pairwise absolute differences in prevalence with corresponding 95% confidence intervals. Statistical significance was assessed using two‐sided tests at *α* = 0.05; no adjustments were made for multiple comparisons. Analyses were conducted using R (version 4.5.2).

## 3 Results

The analytic sample included 61,829 adults receiving LTSS across 27 US states. Overall, 18,004 participants (29%) reported unmet LTSS needs. Compared with those whose needs were met, individuals reporting unmet needs were younger, more likely to have physical or developmental disabilities, more frequently reported fair or poor health, and more often required substantial assistance with self‐care (Table S1 and Table S2).

Weighted prevalence of unmet LTSS needs varied significantly across several strata by demographic, social, health, and functional status (Tables 1 and 2). Unmet needs were higher among younger adults, decreasing from 34.7% in those aged 18–55 years to 23.2% among those aged ≥ 85 years. By race and ethnicity, Asian participants had lower unmet needs (23.2%) compared with Hispanic (31.8%) and other/multiracial individuals (35.6%). Across LTSS programs, substantial differences were observed, with lowest unmet needs among participants in PACE (17.5%) and nursing facilities (21.4%), and highest unmet needs among those receiving services through the OAA (36.1%). Additionally, unmet needs were lower among rural residents (27.5%) compared with metropolitan residents (31.0%). Unmet needs were higher among individuals with mental health diagnoses (36.8% vs. 27.5%) but modestly lower among individuals with an ADRD diagnosis (27.5% vs. 30.5%). Social and living context also showed differences, with higher unmet needs among individuals who were separated or divorced (34.5%) compared with widowed individuals (27.1%), and among those living alone (31.7%) compared with those living with others (24.7%). By care setting, unmet needs were higher in community settings (31.9%) compared with assisted living (21.8%) and nursing homes (22.3%).

**TABLE 1:** Weighted prevalence of person‐reported unmet service needs across demographic strata: The National Core Indicators–Aging & Disability Adult Consumer Survey (2016–2023).

Characteristic	LTSS needs met, *N* = 43,825 weighted % (95% CI)	Unmet LTSS needs, *N* = 18,004 weighted % (95% CI)
Age (years)		
18 to <55	65.3% (63.7, 66.9)	34.7% (33.1, 36.3)
55 to <65	65.9% (64.3, 67.5)	34.1% (32.5, 35.7)
65 to <75	68.5% (67.1, 70.0)	31.5% (30.0, 32.9)
75 to <85	73.2% (71.6, 74.7)	26.8% (25.3, 28.4)
≥ 85	76.8% (74.8, 78.8)	23.2% (21.2, 25.2)
Missing/Unknown	71.0% (66.2, 75.7)	29.0% (24.3, 33.8)
Gender		
Female	69.1% (68.2, 70.0)	30.9% (30.0, 31.8)
Male	71.0% (69.8, 72.2)	29.0% (27.8, 30.2)
Missing/Unknown	72.3% (67.5, 77.0)	27.7% (23.0, 32.5)
Race/Ethnicity		
White	69.9% (69.0, 70.8)	30.1% (29.2, 31.0)
Asian	76.8% (70.9, 82.7)	23.2% (17.3, 29.1)
Black	70.8% (69.2, 72.3)	29.2% (27.7, 30.8)
Hispanic or Latino	68.2% (65.5, 71.0)	31.8% (29.0, 34.5)
Other/Multiracial	64.4% (61.0, 67.8)	35.6% (32.2, 39.0)
Missing/Unknown	65.2% (62.2, 68.1)	34.8% (31.9, 37.8)
ZIP RUCA		
Metropolitan	69.0% (68.2, 69.9)	31.0% (30.1, 31.8)
Micropolitan	69.3% (67.6, 71.0)	30.7% (29.0, 32.4)
Rural	72.5% (69.8, 75.2)	27.5% (24.8, 30.2)
Small town	69.8% (67.0, 72.5)	30.2% (27.5, 33.0)
Missing/Unknown	79.3% (76.8, 81.7)	20.7% (18.3, 23.2)
Waiver		
Medicaid FFS	70.0% (68.9, 71.2)	30.0% (28.8, 31.1)
MLTSS	72.1% (71.2, 73.1)	27.9% (26.9, 28.8)
NF	78.6% (77.1, 80.2)	21.4% (19.8, 22.9)
OAA	63.9% (61.9, 66.0)	36.1% (34.0, 38.1)
PACE	82.5% (79.8, 85.1)	17.5% (14.9, 20.2)
Other	59.7% (56.2, 63.2)	40.3% (36.8, 43.8)
Missing/unknown	68.3% (64.6, 71.9)	31.7% (28.1, 35.4)
Medicare enrollment		
Yes	70.4% (69.5, 71.2)	29.6% (28.8, 30.5)
No	68.1% (66.8, 69.3)	31.9% (30.7, 33.2)
Year		
2016–2017	67.7% (65.5, 69.9)	32.3% (30.1, 34.5)
2017–2018	71.5% (70.3, 72.6)	28.5% (27.4, 29.7)
2018–2019	71.3% (69.8, 72.7)	28.7% (27.3, 30.2)
2021–2022	67.7% (65.6, 69.8)	32.3% (30.2, 34.4)
2022–2023	68.9% (67.5, 70.3)	31.1% (29.7, 32.5)

Abbreviations: CI, Confidence Interval; LTSS, long‐term services and supports; Medicaid FFS, waiver through Medicaid fee‐for‐service system; OAA, Older Americans Act; PACE, Program of All‐inclusive Care for the Elderly; RUCA, Rural–Urban Commuting Area codes.

**TABLE 2:** Weighted prevalence of person‐reported unmet service needs stratified by social, health, and functional characteristics of study participants from the National Core Indicators–Aging & Disability Adult Consumer Survey (2016–2023).

Characteristic	LTSS needs met weighted % (95% CI)	Unmet LTSS needs weighted % (95% CI)
ADRD		
Yes	72.5% (70.3, 74.7)	27.5% (25.3, 29.7)
No	69.5% (68.8, 70.3)	30.5% (29.7, 31.2)
Physical disability		
Yes	66.8% (65.9, 67.7)	33.2% (32.3, 34.1)
No	74.5% (73.4, 75.7)	25.5% (24.3, 26.6)
Developmental disability		
Yes	62.9% (60.0, 65.9)	37.1% (34.1, 40.0)
No	70.3% (69.5, 71.0)	29.7% (29.0, 30.5)
Brain injury		
Yes	62.6% (60.6, 64.6)	37.4% (35.4, 39.4)
No	70.6% (69.9, 71.4)	29.4% (28.6, 30.1)
Mental health diagnosis		
Yes	63.2% (62.0, 64.4)	36.8% (35.6, 38.0)
No	72.5% (71.6, 73.3)	27.5% (26.7, 28.4)
Self‐rated health		
Poor/Fair	63.9% (62.8, 64.9)	36.1% (35.1, 37.2)
Good	76.0% (74.8, 77.2)	24.0% (22.8, 25.2)
Very good/excellent	81.7% (80.1, 83.4)	18.3% (16.6, 19.9)
Missing/unknown	64.8% (59.8, 69.8)	35.2% (30.2, 40.2)
Multiple chronic conditions (> 1)		
Yes	69.3% (68.6, 70.1)	30.7% (29.9, 31.4)
No	76.8% (74.5, 79.1)	23.2% (20.9, 25.5)
Assistance required for self‐care*		
None	72.3% (70.9, 73.6)	27.7% (26.4, 29.1)
Some	70.1% (68.9, 71.2)	29.9% (28.8, 31.1)
A lot	66.9% (65.7, 68.2)	33.1% (31.8, 34.3)
Missing/Unknown	67.9% (61.3, 74.5)	32.1% (25.5, 38.7)
Marital status		
Single	70.2% (68.8, 71.6)	29.8% (28.4, 31.2)
Married/domestic partner	69.3% (67.5, 71.0)	30.7% (29.0, 32.5)
Separated/divorced	65.5% (64.1, 66.9)	34.5% (33.1, 35.9)
Widowed	72.9% (71.4, 74.5)	27.1% (25.5, 28.6)
Missing/unknown	73.3% (71.1, 75.6)	26.7% (24.4, 28.9)
Live with		
Alone	68.3% (67.2, 69.4)	31.7% (30.6, 32.8)
Family	68.7% (67.4, 69.9)	31.3% (30.1, 32.6)
Other	75.3% (73.8, 76.7)	24.7% (23.3, 26.2)
Missing/unknown	74.8% (72.4, 77.3)	25.2% (22.7, 27.6)
Live where		
Community	68.1% (67.3, 69.0)	31.9% (31.0, 32.7)
Assisted living	78.2% (76.1, 80.3)	21.8% (19.7, 23.9)
Nursing home	77.7% (76.1, 79.4)	22.3% (20.6, 23.9)
Other	65.6% (59.2, 71.9)	34.4% (28.1, 40.8)
Missing/unknown	72.2% (69.2, 75.2)	27.8% (24.8, 30.8)

Abbreviations: ADRD, known diagnosis of Alzheimer's disease or related dementias; CI, Confidence Interval; LTSS, long‐term services and supports.

* Response to the question: “How much assistance with self‐care do you generally need? Things like bathing, dressing, going to the bathroom, eating or moving around your home/where you live.”

**FIGURE 1:**
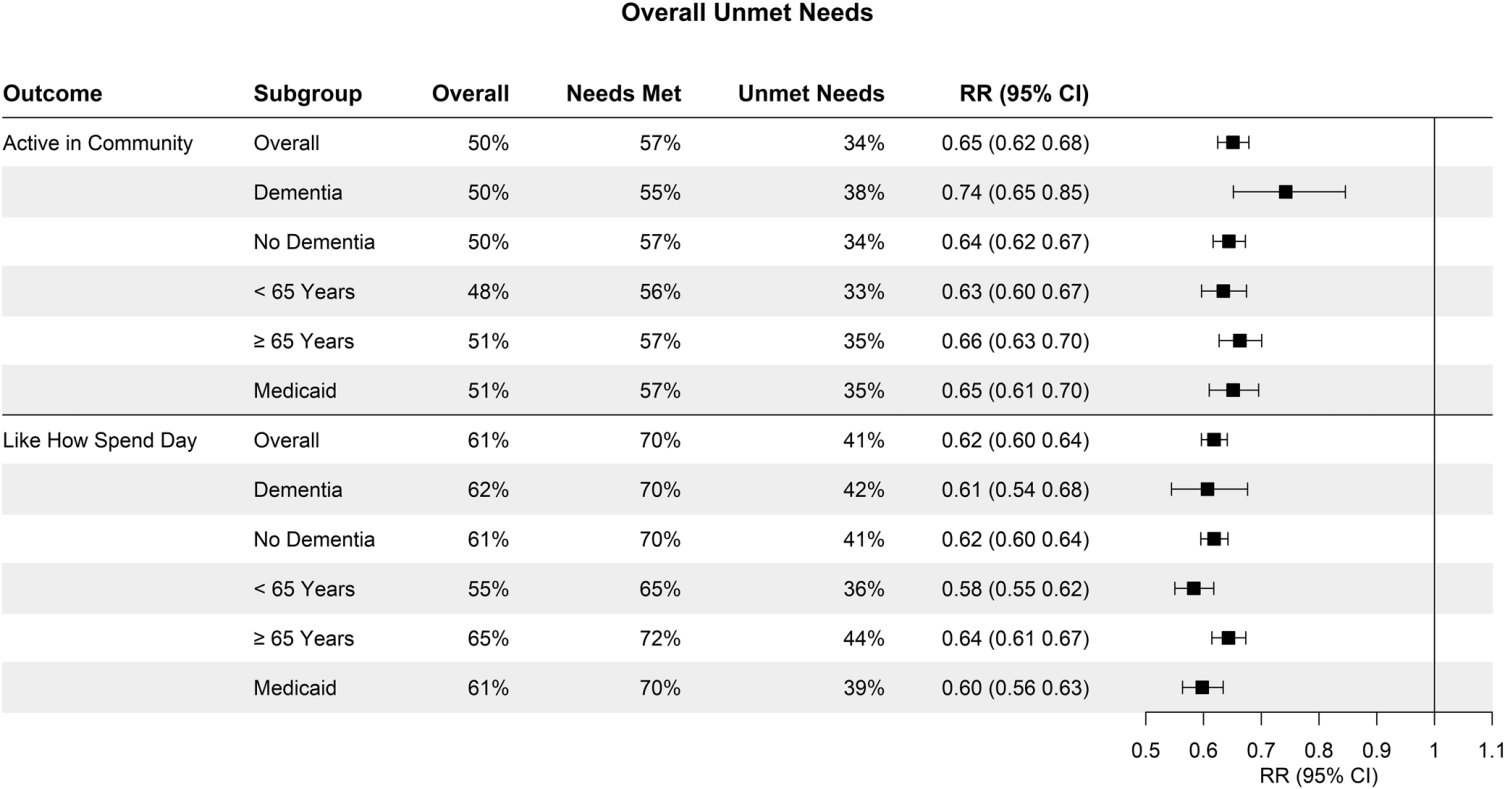
Associations of consumer‐reported unmet service needs with person‐reported outcomes in long‐term services and supports in the United States (2016–2023). Medicaid subgroup with Medicaid waiver as the primary source of LTSS funding; PR‐ prevalence ratio adjusted for age, sex, race and ethnicity, disability type, mental‐health and physical‐health diagnoses, self‐rated health, level of assistance needed for self‐care, marital status, living arrangement, residence setting, LTSS funding program, Medicare enrollment, proxy response, survey year, and state of residence; computed by applying survey weights to generate population‐representative estimates; rates are presented as unweighted frequency/number (weighted %).

In weighted unadjusted analyses (Figure 1), respondents with unmet needs were 23 percentage points less likely to report being active in the community (34% vs. 57%) and 29 percentage points less likely to report satisfaction with how they spend their day (41% vs. 70%). In weighted regression analyses, unmet LTSS needs were associated with poorer person‐reported outcomes (Figure 1 and Table S3). Compared with those whose needs were met, individuals with unmet needs were 35% less likely to report being active in the community (PR, 0.65; 95% CI, 0.62–0.68) and 38% less likely to be satisfied with how they spend their day (PR, 0.62; 95% CI, 0.60–0.64). Results were consistent across prespecified subgroups, including age strata, dementia status, and Medicaid waiver enrollment (Figure 1). Associations persisted in sensitivity analyses excluding consumers in nursing facilities (Figure S2) and in the subgroup of consumers in nursing facilities (Figure S3). Findings were similar in sensitivity analyses using augmented inverse probability weighting to compute doubly‐robust estimators (Table S4).

**FIGURE 2:**
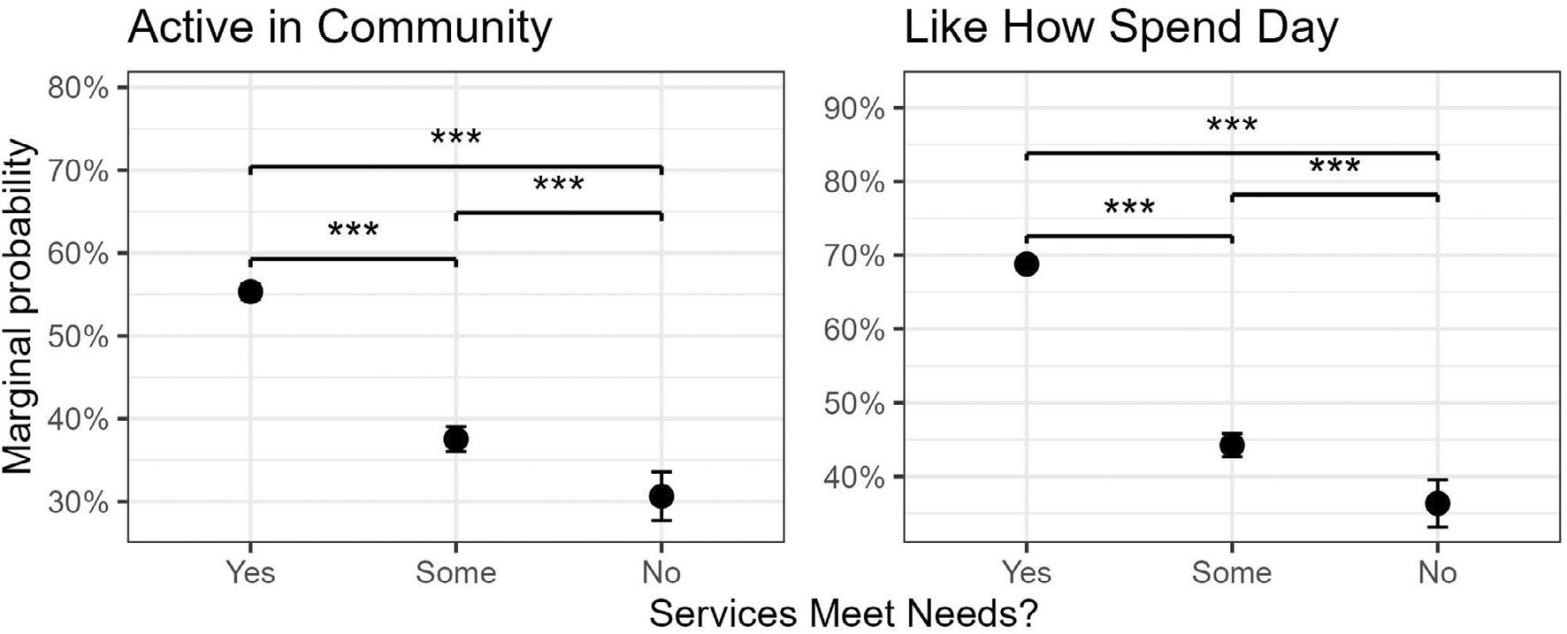
Associations of degree of unmet service needs with person‐reported outcomes in long‐term services and supports in the United States (2016–2023) ***p < 0.001. Estimates of percent prevalence and risk difference are presented in Table 2.

In post hoc analyses, greater degrees of unmet LTSS needs were associated with progressively worse person‐reported QoL outcomes (Table 3; Figure 2). Compared with participants reporting no unmet needs, those reporting some unmet needs had lower prevalence of being active in the community (PR, 0.68; 95% CI, 0.65–0.71) and liking how they spend their day (PR, 0.64; 95% CI, 0.62–0.67). Marginal prevalence estimates demonstrated graded absolute differences, with the subgroup of participants reporting unmet needs having 24.7% lower community participation and 32.4% lower satisfaction with daily activities compared with the subgroup reporting no unmet needs (Table 3).

**TABLE 3:** Associations between degree of unmet service needs and person‐reported outcomes and healthcare utilization in long‐term services and supports in the United States (2016–2023): Post hoc dose–response analyses.

Unmet LTSS needs	Active in community	Like how spend day
Conditional prevalence ratios (95% CI)		
No	Reference	Reference
Some	0.68 (0.65, 0.71) ***	0.64 (0.62, 0.67) ***
Yes	0.55 (0.50, 0.61) ***	0.53 (0.48, 0.58) ***
Marginal probabilities, % prevalence (95% CI)		
Yes	30.6% (27.7%, 33.5%)	36.4% (33.2%, 39.6%)
Some	37.5% (36.0%, 39.0%)	44.3% (42.7%, 45.8%)
No	55.3% (54.4%, 56.3%)	68.8% (68.0%, 69.6%)
Pairwise contrasts, differences in % marginal prevalences (95% CI)		
Some–yes	6.9% (3.6%, 10.2%) ***	7.9% (4.3%, 11.4%) ***
No–yes	24.7% (21.6%, 27.8%) ***	32.4% (29.1%, 35.7%) ***
No–some	17.8% (16.0%, 19.6%) ***	24.5% (22.7%, 26.3%) ***

*Note:* ***p‐value < 0.001, Estimates are from regression models adjusted for age, sex, race and ethnicity, disability type, mental‐health and physical‐health diagnoses, self‐rated health, level of assistance needed for self‐care, marital status, living arrangement, residence setting, LTSS funding program, Medicare enrollment, proxy response, survey year, and state of residence; computed by applying survey weights to generate population‐representative estimates.

Abbreviations: CI, Confidence Interval; LTSS, long‐term services and supports.

## 4 Discussion

In this large, multi‐state sample of 61,829 individuals receiving LTSS, we found that consumer‐reported unmet LTSS needs identified a population at markedly higher likelihood of adverse outcomes. Individuals reporting unmet LTSS needs were significantly less likely to report community engagement or satisfaction with their daily activities. These associations were robust across strata by age groups and dementia status, and among the subgroup of Medicaid waiver beneficiaries, underscoring the consistency of unmet LTSS needs as a marker of vulnerability. The associations persisted in sensitivity analyses excluding nursing home residents. Furthermore, post hoc analyses showed a potential dose–response association between increasing degree of unmet LTSS needs and poorer outcomes.

Social isolation is a silent epidemic among older adults which can lead to poorer QoL and adverse health outcomes such as depression [9, 10, 11, 12]. In our analyses, we found that greater unmet LTSS needs are associated with a lower probability of being active in the community, which in turn could lead to social isolation [10, 13, 14]. Furthermore, we found that greater unmet LTSS needs are associated with lower satisfaction with how one spends one's day, which could contribute to worsening mental and physical health [15, 16]. Future work should confirm whether addressing unmet LTSS needs may help mitigate the risk of social isolation among older adults and adults with disabilities [13].

LTSS includes a patchwork of disparate services making it complex to navigate [1, 2, 3]. Identifying a high prevalence of unmet LTSS needs raises important practical questions about what it would mean to subsequently address those needs at scale within a complex and fragmented LTSS ecosystem. Unmet needs likely encompass multiple domains, including assistance with activities of daily living, transportation, social engagement, caregiver support, and coordination of medical and social services [3, 17]. However, identifying individuals with unmet needs does not, in itself, resolve the underlying gaps in service availability, access, and coordination. Given the high prevalence of unmet needs observed in this study, attempting to meet these needs would require substantial expansion and integration of LTSS infrastructure across settings, programs, and funding streams [3, 17, 18, 19]. In this context, systematically screening for unmet LTSS needs in routine practice may serve less as a tool for individual‐level intervention and more as an indicator of broader system‐level deficiencies, highlighting the scale of unmet demand and the need for coordinated policy and investment to address these gaps [17, 18, 19]. Another solution may be to expand case management and care coordination services for LTSS consumers. Prior literature suggests that programs like PACE and managed care, which intrinsically mandate service coordination, are associated with lower unmet needs compared with traditional Medicaid fee‐for‐service systems [19]. Evidence from multiple LTSS and care transitions programs demonstrates that coordinated care, including structured case management, proactive service linkage, and targeted problem‐solving, can reduce acute care utilization and improve functional and psychosocial outcomes [19, 20, 21, 22, 23]. Finally, identifying and addressing unmet LTSS needs may also impact physician wellbeing as previous studies indicate an association between clinic's capacity to address social needs and physician burnout and satisfaction [24, 25].

Policy implications are equally salient. State Medicaid agencies face recurring pressure to constrain LTSS and home‐ and community‐based services spending despite rising demand [1, 2]. We found that associations of unmet LTSS needs with poorer QoL‐related outcomes and greater healthcare utilization persisted in the subpopulation with Medicaid as the primary source of LTSS funding. Our findings highlight the potential downstream consequences of unmet needs among LTSS users, suggesting that insufficient access to necessary supports may generate greater reliance on costly emergency and inpatient care [1, 2]. These results reinforce the importance of maintaining and expanding LTSS infrastructure, strengthening service coordination capacity, and ensuring adequate provider networks to meet the needs of aging and disabled populations [26, 27, 28, 29, 30]. Additionally, the high prevalence of unmet needs observed across diverse settings, programs, and populations suggests that gaps in care are not isolated to specific services but instead reflect broader structural shortcomings of the current LTSS system [1, 2, 3]. Given the broad definition of LTSS used in this study including medical, personal, and social supports, the persistence of unmet needs indicates that the existing patchwork of programs, funding streams, and eligibility pathways may be insufficiently coordinated and inadequately resourced to meet the needs of LTSS users [3, 17, 18, 19]. From a policy perspective, these results underscore the need not only to expand LTSS capacity, particularly home‐ and community‐based services, but also to improve integration across programs, align incentives across payers, and strengthen the workforce required to deliver and coordinate services [19]. Taken together, these findings suggest that addressing unmet LTSS needs will likely require system‐level reforms rather than incremental changes within individual programs.

This study has several strengths, including its use of a large, diverse, nationally pooled dataset, population‐representative estimates generated by survey weights, reliance on standardized, person‐reported measures, and inclusion of outcomes that reflect both health care utilization and everyday well‐being. Several limitations exist. The cross‐sectional survey design precludes drawing causal inferences. Self‐reported outcomes may be susceptible to recall bias, which would likely lead to non‐differential misclassification biasing results toward the null. LTSS program structures vary between states; to address this, we adjusted for state of residence. There is potential for residual and unmeasured confounding despite extensive covariate adjustment and sensitivity analyses using augmented inverse probability weighting to compute doubly‐robust estimators. Nonetheless, the consistency and magnitude of the associations observed across multiple strata support the importance of unmet LTSS needs as a measurable, policy‐relevant signal of risk within this population.

## 5 Conclusion

In this multistate study of older adults receiving publicly‐funded LTSS, unmet service needs were common and varied across settings, programs, and populations. These unmet needs were consistently associated with poorer person‐reported outcomes. Together, these findings highlight substantial system‐level gaps in the current LTSS landscape and underscore the need for coordinated investments and structural reforms to better meet the needs of those relying on LTSS.

## Author Contributions

**R.R.P.:** study concept and design, analysis, interpretation, preparation of first draft, and final approval. **J.M.W.:** study design, analysis, interpretation, critically reviewing and revising draft, and final approval. **N.U.S.**, **A.A.P.**, **B.W.L.**, and **C.D.F.:** analysis, interpretation, critically reviewing draft, and final approval. **S.G.**, **E.J.**, and **T.S.:** study design, data acquisition, analysis, interpretation, critically reviewing draft, and final approval.

## Funding

The analyses in this manuscript were supported by the National Institute on Aging of the National Institutes of Health under Award Numbers RF1AG069771 and 3RF1AG069771‐01S1.

## Disclosure

The funder/sponsor of this work had no role in the design or conduct of this study.

## Conflicts of Interest

Dr. Jutkowitz is a co‐founder and on the board of directors of Plans4Care Inc., a digital health company that provides personalized dementia care on‐demand. The remaining authors have no conflicts of interest to disclose.

## Supporting information


**Data S1:** Supplemental Methods: Description of the NCI‐AD survey and survey weights.
**Table S1:** Summary of demographics of study participants from the National Core Indicators–Aging & Disability Adult Consumer Survey (2016–2023).
**Table S2:** Summary of social, health, and functional characteristics of study participants from the National Core Indicators–Aging & Disability Adult Consumer Survey (2016–2023).
**Table S3:** Associations of consumer‐reported unmet service needs with person‐reported outcomes in long‐term services and supports in the United States (2016–2023): Summary of estimated regression models for the prevalence ratio of each outcome variable.
**Table S4:** Associations of consumer‐reported unmet service needs with person‐reported outcomes in long‐term services and supports in the United States (2016–2023): Estimated using augmented inverse probability weighting.
**Figure S1:** Participant inclusion flow diagram.
**Figure S2:** Associations of consumer‐reported unmet service needs with person‐reported outcomes in long‐term services and supports in the United States (2016–2023): Excluding respondent from nursing facilities. Prevalence ratio adjusted for age, sex, race and ethnicity, disability type, mental‐health and physical‐health diagnoses, self‐rated health, level of assistance needed for self‐care, marital status, living arrangement, residence setting, LTSS funding program, Medicare enrollment, proxy response, survey year, and state of residence; computed by applying survey weights to generate population‐representative estimates.
**Figure S3:** Associations of consumer‐reported unmet service needs with person‐reported outcomes in long‐Term SERVICES and supports in the United States (2016–2023): Including only respondent from nursing facilities. Prevalence ratio adjusted for age, sex, race and ethnicity, disability type, mental‐health and physical‐health diagnoses, self‐rated health, level of assistance needed for self‐care, marital status, Medicare enrollment, proxy response, survey year, and state of residence; computed by applying survey weights to generate population‐representative estimates.
