# Supplementary material for: Prevalence of Unmet Service Needs and Associated Person‐Reported Outcomes in Long‐Term Services and Supports in the United States
Source: J Am Geriatr Soc. 2026 Jun 19;74(8):2235–44. doi: 10.1111/jgs.70551 (PMC13395546; doi:10.1111/jgs.70551)
Supplement: Supplementary file 1 [file JGS-74-2235-s001.pdf]

Online Supplement

Supplemental Methods

The National Core Indicators-Aging & Disability (NCI-AD) Adult Consumer Survey (ACS).

The NCI-AD ACS consists of a consumer-section which asks long term care supports and services (LTSS) consumers questions to provide a comprehensive assessment of care experiences, satisfaction with services, quality of life, health outcomes, and the effectiveness of LTSS in promoting independence and autonomy; and a Background Information section which pulls data primarily from administrative records described below. Prior to the COVID-19 pandemic (till the year 2019), the surveys were conducted in-person. Beginning in the 2021-22 survey year, based on a successful pilot validation study, the NCI-AD allowed states to use remote methods such as telephone to conduct the ACS. Proxy respondents are allowed by survey administrators, for select items if the consumer cannot respond to the consumer-section of the survey on their own behalf. The sampling frame is determined by the participating states as follows. A probabilistic sampling technique is used by participating states to select participants from the eligible population for their services, often for specific funding programs, aiming for a margin of error no greater than 5% at a 95% confidence level. Before conducting interviews, comprehensive background information about survey participants including demographics, personal traits, legal status, services received, and clinical diagnoses are systematically gathered from administrative records and then linked to the survey responses. This linkage forms a complete data profile for each participant, combining both administrative and survey data. The Background Information section of the survey, which includes details such as race, gender, mental health conditions, Alzheimer's disease, related dementias, and disabilities, is typically sourced from state administrative records like case management, managed care, and Medicaid billing data. Administrative records are crucial for identifying service types, often obtained from the Medicaid Management Information System, rather than relying on self-reported data.

Sample selection usually begins within 3-4 months of the commencement of the annual survey wave. However, due to the extended data collection period, individuals may be surveyed up to 8 months after the sample is selected. The NCI-AD background information section depends on state administrative records for demographic and service-related information. When these records are unavailable, participants may be directly asked at the end of the survey. Certain subsections of the "Background Information" section, such as primary funding for LTSS, the primary program, services received, duration of participation, self-directed support, and legal guardianship status, require data to be collected from administrative records. To qualify for the survey, individuals must be receiving at least one active service (e.g., personal care, homemaker, transportation) at least twice a week for about three months. Active LTSS use is a necessary condition for participation in the survey.

The NCI-AD ACS Survey Weights

States determine their sampling frame and several participating states intentionally oversampled certain LTSS program groups, meaning that some programs accounted for a larger share of the survey sample than they do in the underlying service population. To correct for this imbalance, the NCI-AD team created analytic weights so that state-level and program-level estimates reflect the true distribution of individuals receiving services. These weights were derived by comparing each program's proportion in the sample with its proportion in the full state population eligible for the survey. In the same way, the overall NCI-AD averages/estimates incorporate weighting to account for differences across states in both sample size and the number of residents eligible for inclusion. Thus,

each state contributes to national estimates in proportion to the size of its target LTSS population, adjusted for the state's sampling strategy. In each survey wave, before data collection begins, the NCI-AD project team collaborates with each state to define mutually exclusive program populations and to determine the number of eligible service recipients within each program. These program population counts, combined with the corresponding sample counts, are used to construct all weighting variables applied in the present analyses. The weights are constructed by the analysis team at the NCI-AD ACS project team led by the Human Services Research Institute (Cambridge, Massachusetts) and ADvancing States.

Sensitivity Analyses: Augmented Inverse Probability Weighting

To assess the sensitivity of the primary findings to potential outcome model misspecification arising from covariate imbalance between exposure groups, we conducted a supplementary analysis using a survey-weighted doubly robust estimator targeting marginal risk ratios. This analysis was implemented using the same analytic sample, covariate set, and survey design as the primary models, with marginal risks estimated via influence-function–based linearization and confidence intervals obtained from a design-based variance estimator treating nuisance parameters as fixed, yielding conservative inference.

Supplemental Results

Supplemental Table S1. Summary of demographics of study participants from the National Core Indicators- Aging & Disability Adult Consumer Survey (2016-2023).

**Characteristic**	**Overall**, N = 61,829	**LTSS Needs Met**, N = 43,825	**Unmet LTSS Needs**, N = 18,004
Age (years)			
[18,55)	12306 (15%)	8167 (14%)	4139 (18%)
[55,65)	12516 (19%)	8476 (18%)	4040 (22%)
[65,75)	14555 (26%)	10166 (25%)	4389 (26%)
[75,85)	12238 (21%)	9134 (22%)	3104 (19%)
[85,Inf)	9150 (17%)	7102 (18%)	2048 (13%)
Missing/Unknown	1064 (1.9%)	780 (1.9%)	284 (1.9%)
Gender			
Female	40015 (66%)	28323 (66%)	11692 (68%)

Male	21113 (33%)	15012 (33%)	6101 (31%)
Missing/Unknown	701 (1.0%)	490 (1.0%)	211 (0.9%)
Race/Ethnicity			
White	37015 (60%)	26473 (61%)	10542 (60%)
Asian	1706 (3.4%)	1238 (3.6%)	468 (2.7%)
Black	13211 (21%)	9360 (22%)	3851 (21%)
Hispanic or Latino	4232 (6.1%)	2935 (6.0%)	1297 (6.4%)
Other/Multiracial	2252 (3.3%)	1487 (3.1%)	765 (3.8%)
Missing/Unknown	3413 (5.3%)	2332 (4.9%)	1081 (6.2%)
ZIP RUCA			
Metropolitan	42022 (70%)	29372 (70%)	12650 (72%)
Micropolitan	9189 (13%)	6605 (13%)	2584 (14%)
Rural	2960 (4.3%)	2166 (4.4%)	794 (3.8%)
Small town	5378 (7.6%)	3933 (7.7%)	1445 (7.5%)
Missing/Unknown	2280 (4.4%)	1749 (4.9%)	531 (3.1%)
Waiver			
Medicaid	28490 (44%)	20062 (44%)	8428 (44%)
MLTSS	16489 (23%)	11800 (24%)	4689 (21%)
NF	5349 (9.8%)	4165 (11%)	1184 (6.6%)
OAA	6337 (19%)	3991 (17%)	2346 (22%)
PACE	1296 (0.4%)	1074 (0.5%)	222 (0.3%)
Other	2893 (3.3%)	2071 (2.9%)	822 (4.4%)
Missing/Unknown	975 (0.9%)	662 (0.9%)	313 (0.9%)
Medicare	43570 (73%)	31231 (74%)	12339 (72%)
Proxy			
Proxy	10725 (16%)	7854 (17%)	2871 (14%)
Not Proxy	51072 (84%)	35950 (83%)	15122 (86%)

Missing/Unknown	32 (<0.1%)	21 (<0.1%)	11 (<0.1%)
Year			
2016	8444 (15%)	5806 (15%)	2638 (17%)
2017	16650 (27%)	11970 (27%)	4680 (25%)
2018	13329 (22%)	9599 (23%)	3730 (21%)
2021	11204 (17%)	7761 (17%)	3443 (19%)
2022	12202 (19%)	8689 (19%)	3513 (19%)

Note: Values are weighted % (95% confidence interval). ADRD- Alzheimer’s disease and related dementias; LTSS- long-term services and supports; NF- Nursing Facility; OAA- Older Americans Act; PACE- Program of All-inclusive Care for the Elderly; RUCA- Rural-Urban Commuting Area codes

Supplemental Table S2. **Summary of social, health, and functional characteristics of study participants from the National Core Indicators- Aging & Disability Adult Consumer Survey (2016-2023).**

**Characteristic**	**Overall**, N = 61,829	**LTSS Needs Met**, N = 43,825	**Unmet LTSS Needs**, N = 18,004
ADRD	8447 (13%)	6297 (13%)	2150 (11%)
Physical Disability	38199 (61%)	26135 (59%)	12064 (67%)
Developmental Disability	5664 (8.6%)	3756 (7.8%)	1908 (11%)
Brain Injury	7653 (12%)	5021 (11%)	2632 (15%)
Mental Health Diagnosis	18908 (29%)	12415 (27%)	6493 (35%)
Self-Rated Health			
Poor/Fair	33275 (56%)	21869 (52%)	11406 (66%)
Good	18643 (29%)	14100 (32%)	4543 (24%)
Very Good/Excellent	8974 (13%)	7257 (15%)	1717 (8.2%)
Missing/Unknown	937 (1.4%)	599 (1.3%)	338 (1.6%)
Assistance Required for Self-Care			
None	14215 (25%)	10419 (26%)	3796 (24%)
Some	22431 (36%)	15946 (36%)	6485 (36%)
A lot	24676 (38%)	17113 (37%)	7563 (39%)
Missing/Unknown	507 (0.8%)	347 (0.8%)	160 (0.8%)
Marital Status			
Single	16335 (24%)	11370 (24%)	4965 (24%)
Married/Domestic Partner	10379 (16%)	7335 (16%)	3044 (16%)
Separated/Divorced	14926 (25%)	10148 (24%)	4778 (29%)
Widowed	14491 (26%)	10811 (27%)	3680 (23%)
Missing/Unknown	5698 (8.7%)	4161 (9.2%)	1537 (7.5%)
Live With			

Alone	24655 (44%)	17153 (43%)	7502 (47%)
Family	21847 (35%)	15058 (34%)	6789 (37%)
Other	11104 (15%)	8541 (16%)	2563 (11%)
Missing/Unknown	4223 (6.6%)	3073 (7.1%)	1150 (5.6%)
Live Where			
Community	47270 (79%)	32649 (77%)	14621 (84%)
Assisted Living	6371 (7.3%)	5049 (8.2%)	1322 (5.2%)
Nursing Home	5817 (10%)	4505 (11%)	1312 (6.9%)
Other	645 (1.0%)	414 (0.9%)	231 (1.1%)
Missing/Unknown	1726 (2.7%)	1208 (2.7%)	518 (2.6%)
Proxy			
Proxy	10725 (16%)	7854 (17%)	2871 (14%)
Not Proxy	51072 (84%)	35950 (83%)	15122 (86%)
Missing/Unknown	32 (<0.1%)	21 (<0.1%)	11 (<0.1%)
Year			
2016	8444 (15%)	5806 (15%)	2638 (17%)
2017	16650 (27%)	11970 (27%)	4680 (25%)
2018	13329 (22%)	9599 (23%)	3730 (21%)
2021	11204 (17%)	7761 (17%)	3443 (19%)
2022	12202 (19%)	8689 (19%)	3513 (19%)

Values are N (Weighted %). ADRD- Alzheimer’s disease and related dementias; LTSS- long-term services and supports

Supplemental Table S3. Associations of Consumer-Reported Unmet Service Needs With Person-Reported Outcomes in Long-Term Services and Supports in the United States (2016-2023): Summary of estimated regression models for the prevalence ratio of each outcome variable.

	Active in Community (N = 48,728)	Like How Spend Day (N = 49,027)
**Characteristic**	**PR (95% CI)**	**PR (95% CI)**
Unmet Needs	0.65 (0.62, 0.68)***	0.62 (0.60, 0.64)***
Age (years)		
[0,55)	—	—
[55,65)	0.98 (0.94, 1.03)	1.04 (1.00, 1.09)*
[65,75)	1.01 (0.96, 1.06)	1.12 (1.08, 1.17)***
[75,85)	1.01 (0.95, 1.07)	1.13 (1.08, 1.18)***
[85,Inf)	1.05 (0.98, 1.13)	1.18 (1.12, 1.24)***
Missing/Unknown	0.98 (0.87, 1.11)	0.99 (0.86, 1.12)
Gender		
Female	—	—
Male	1.04 (1.01, 1.08)*	0.99 (0.97, 1.02)
Missing/Unknown	1.16 (1.01, 1.33)*	1.20 (1.04, 1.38)*
Race/Ethnicity		
White	—	—
Asian	0.86 (0.71, 1.04)	0.99 (0.90, 1.09)
Black or African-American	1.05 (1.01, 1.10)*	1.05 (1.02, 1.08)**
Hispanic or Latino	0.91 (0.84, 0.99)*	1.09 (1.04, 1.15)***
Other/Multiracial	1.01 (0.94, 1.09)	1.04 (0.98, 1.10)
Missing/Unknown	1.01 (0.95, 1.08)	1.01 (0.95, 1.07)
ADRD	1.03 (0.98, 1.09)	1.00 (0.96, 1.04)
Physical Disability	0.95 (0.92, 0.99)*	0.98 (0.95, 1.01)

Characteristic	Active in Community (N = 48,728)<br>PR (95% CI)	Like How Spend Day (N = 49,027)<br>PR (95% CI)
Developmental Disability	1.11 (1.05, 1.19)***	1.05 (1.00, 1.11)
Brain Injury	0.99 (0.94, 1.04)	0.97 (0.93, 1.01)
Mental Health Diagnosis	0.93 (0.90, 0.96)***	0.93 (0.90, 0.96)***
Self Rated Health		
Poor/Fair	—	—
Good	1.23 (1.19, 1.27)***	1.21 (1.18, 1.25)***
Very Good/Excellent	1.34 (1.28, 1.40)***	1.33 (1.29, 1.37)***
Missing/Unknown	1.08 (0.95, 1.23)	1.04 (0.91, 1.18)
Assistance Required for Self Care		
None	—	—
Some	0.96 (0.92, 0.99)*	0.96 (0.93, 0.99)**
A lot	0.86 (0.83, 0.90)***	0.94 (0.91, 0.97)***
Missing/Unknown	0.91 (0.73, 1.13)	0.89 (0.79, 1.01)
Chronic Diagnoses	0.88 (0.84, 0.92)***	0.96 (0.93, 1.0)*
Marital Status		
Single	—	—
Married/Domestic Partner	1.01 (0.95, 1.06)	0.95 (0.91, 0.99)**
Separated/Divorced	1.01 (0.96, 1.06)	0.92 (0.89, 0.95)***
Widowed	1.04 (0.98, 1.10)	0.97 (0.93, 1.00)
Missing/Unknown	0.99 (0.93, 1.05)	0.89 (0.84, 0.94)***
Live With		
Alone	—	—

Characteristic	Active in Community (N = 48,728)<br>PR (95% CI)	Like How Spend Day (N = 49,027)<br>PR (95% CI)
Family	1.02 (0.98, 1.06)	1.05 (1.02, 1.08)**
Other	1.01 (0.96, 1.07)	0.99 (0.94, 1.03)
Missing/Unknown	0.98 (0.91, 1.05)	1.00 (0.94, 1.06)
Live Where		
Community	—	—
Assited Living	1.08 (1.02, 1.13)**	1.03 (0.99, 1.08)
Nursing Home	0.95 (0.86, 1.05)	0.97 (0.90, 1.06)
Other	1.07 (0.92, 1.24)	1.00 (0.89, 1.11)
Missing/Unknown	1.11 (1.02, 1.21)*	1.01 (0.93, 1.10)
ZIP RUCA		
Metropolitan	—	—
Micropolitan	1.04 (1.00, 1.08)	1.01 (0.97, 1.04)
Rural	1.08 (1.02, 1.15)*	1.03 (0.98, 1.08)
Small town	1.03 (0.98, 1.09)	1.00 (0.95, 1.04)
Missing/Unknown	1.19 (1.12, 1.27)***	1.00 (0.94, 1.06)
Waiver		
Medicaid	—	—
MLTSS	1.04 (0.99, 1.09)	1.00 (0.97, 1.04)
NF	1.18 (1.08, 1.29)***	0.92 (0.85, 0.99)*
OAA	1.03 (0.97, 1.10)	1.02 (0.97, 1.07)
PACE	1.13 (1.03, 1.24)*	1.03 (0.97, 1.09)
Other	0.99 (0.92, 1.08)	0.98 (0.92, 1.04)
Missing/Unknown	0.86 (0.79, 0.95)**	0.92 (0.85, 0.99)*

Characteristic	Active in Community (N = 48,728)<br>PR (95% CI)	Like How Spend Day (N = 49,027)<br>PR (95% CI)
Medicare	0.98 (0.95, 1.01)	1.00 (0.97, 1.04)

*p<0.05; **p<0.01; ***p<0.001. Models are adjusted for survey year and state id

ADRD- Alzheimer's disease and related dementias; CI- Confidence Interval; LTSS- long-term services and supports; NF- Nursing Facility; OAA- Older Americans Act; PACE- Program of All-inclusive Care for the Elderly; PR- Prevalence Ratio; RUCA- Rural-Urban Commuting Area codes

Supplemental Table S4: Associations of Consumer-Reported Unmet Service Needs With Person-Reported Outcomes in Long-Term Services and Supports in the United States (2016-2023): Estimated Using Augmented Inverse Probability Weighting

	Marginal Prevalence Ratio (95% CI)	
Outcome	Parametric	Nonparametric
Active in Community	0.65 (0.63, 0.68)	0.66 (0.62, 0.69)
Like How Spend Day	0.63 (0.60, 0.65)	0.63 (0.61, 0.66)

Supplemental Figure S1. Participant Inclusion Flow Diagram.

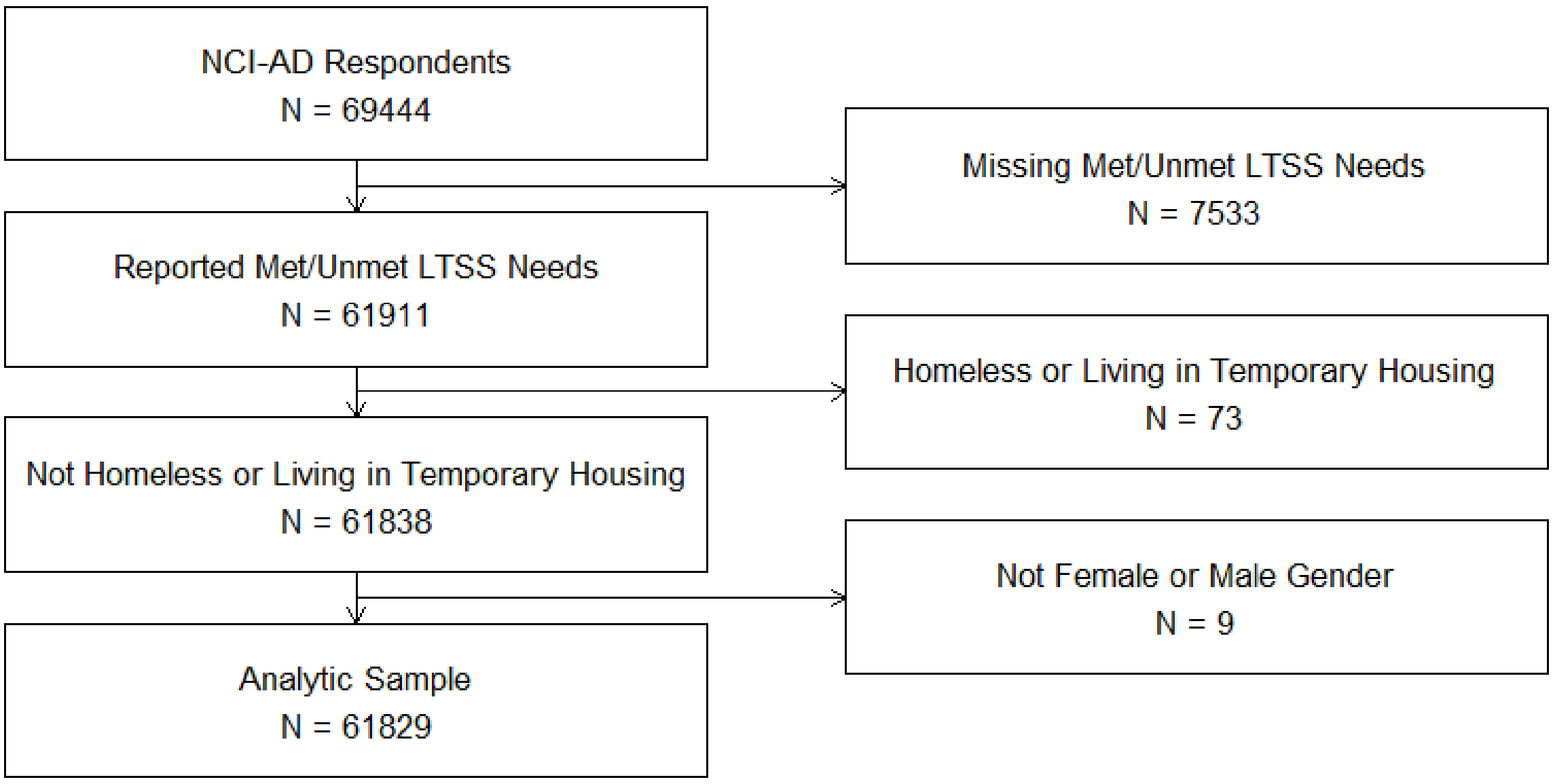

Supplemental Figure S2. Associations of Consumer-Reported Unmet Service Needs With Person-Reported Outcomes in Long-Term Services and Supports in the United States (2016-2023): Excluding Respondent from Nursing Facilities.

Overall Unmet Needs

Outcome	Subgroup	Overall	Needs Met	Unmet Needs	RR (95% CI)
Active in Community	Overall	50%	57%	34%	0.65 (0.62 0.68)
	Dementia	50%	55%	38%	0.74 (0.65 0.85)
	No Dementia	50%	57%	34%	0.64 (0.62 0.67)
	< 65 Years	48%	56%	33%	0.63 (0.60 0.67)
	≥ 65 Years	51%	57%	35%	0.66 (0.63 0.70)
	Medicaid	51%	57%	35%	0.65 (0.61 0.70)
Like How Spend Day	Overall	61%	70%	41%	0.62 (0.60 0.64)
	Dementia	62%	70%	42%	0.61 (0.54 0.68)
	No Dementia	61%	70%	41%	0.62 (0.60 0.64)
	< 65 Years	55%	65%	36%	0.58 (0.55 0.62)
	≥ 65 Years	65%	72%	44%	0.64 (0.61 0.67)
	Medicaid	61%	70%	39%	0.60 (0.56 0.63)

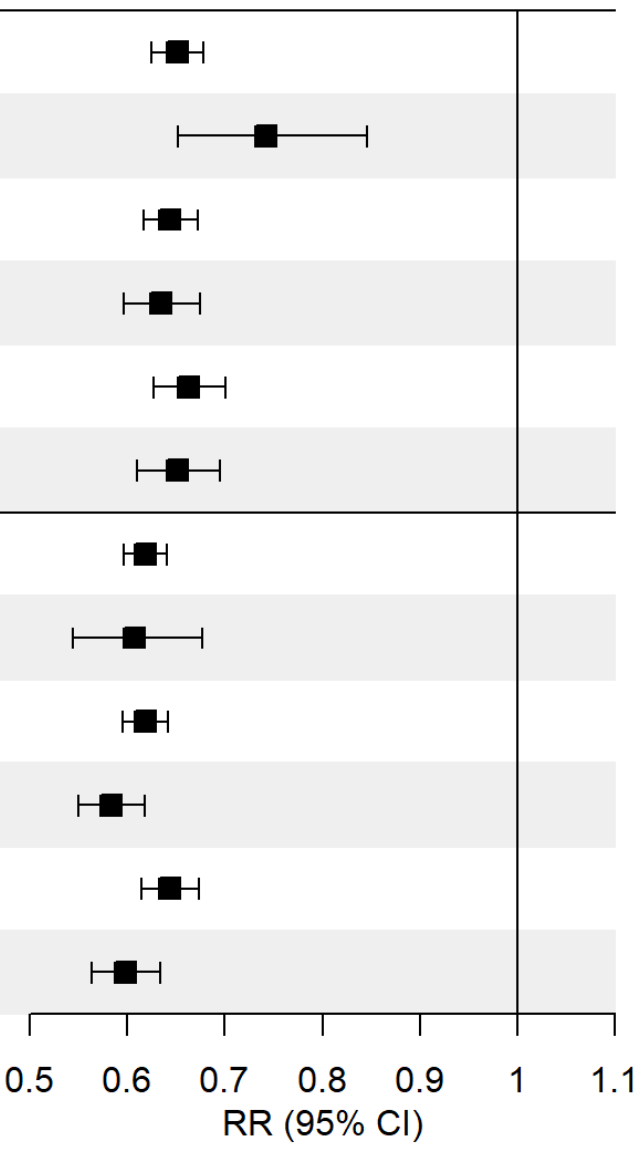

Note: Prevalence ratio adjusted for age, sex, race and ethnicity, disability type, mental-health and physical-health diagnoses, self-rated health, level of assistance needed for self-care, marital status, living arrangement, residence setting, LTSS funding program, Medicare enrollment, proxy response, survey year, and state of residence; computed by applying survey weights to generate population-representative estimates.

Supplemental Figure S3. Associations of Consumer-Reported Unmet Service Needs With Person-Reported Outcomes in Long-Term Services and Supports in the United States (2016-2023): Including Only Respondent from Nursing Facilities.

Overall Unmet Needs: Only Nursing Home Residents

Outcome	Subgroup	Overall	Needs Met	Unmet Needs	RR (95% CI)
Active in Community	Overall	56%	61%	36%	0.62 (0.54 0.70)
	Dementia	57%	61%	38%	0.73 (0.64 0.83)
	No Dementia	56%	61%	36%	0.64 (0.62 0.67)
Like How Spend Day	Overall	57%	65%	29%	0.46 (0.40 0.54)
	Dementia	58%	66%	25%	0.61 (0.54 0.68)
	No Dementia	57%	65%	29%	0.62 (0.60 0.64)

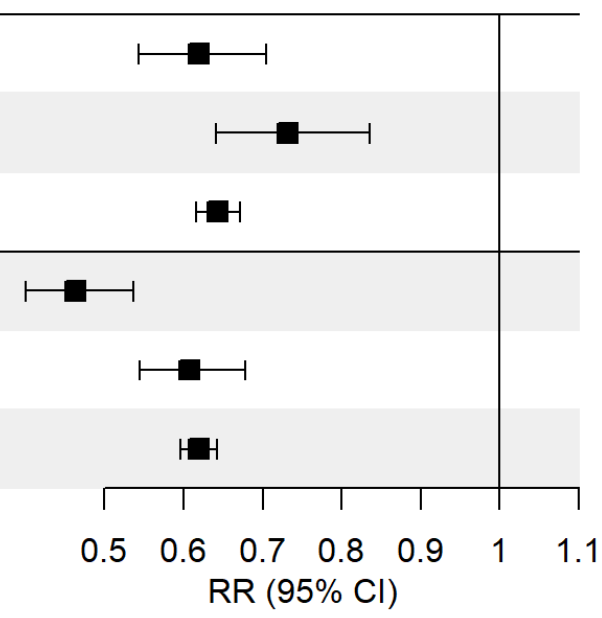

Note: Prevalence ratio adjusted for age, sex, race and ethnicity, disability type, mental-health and physical-health diagnoses, self-rated health, level of assistance needed for self-care, marital status, Medicare enrollment, proxy response, survey year, and state of residence; computed by applying survey weights to generate population-representative estimates.
